# Prevention of Mental Health Disorders Using Internet- and Mobile-Based Interventions: A Narrative Review and Recommendations for Future Research

**DOI:** 10.3389/fpsyt.2017.00116

**Published:** 2017-08-10

**Authors:** David Daniel Ebert, Pim Cuijpers, Ricardo F. Muñoz, Harald Baumeister

**Affiliations:** ^1^Clinical Psychology and Psychotherapy, Friedrich-Alexander University of Erlangen-Nürnberg, Erlangen, Germany; ^2^Department of Clinical, Neuro and Developmental Psychology, Vrije Universiteit Amsterdam, Amsterdam, Netherlands; ^3^Palo Alto University, Palo Alto, CA, United States; ^4^University of California, San Francisco, San Francisco, CA, United States; ^5^Department of Clinical Psychology and Psychotherapy, University of Ulm, Ulm, Germany

**Keywords:** mental health, self-help, e-health, m-Health, Internet interventions, depression, anxiety, prevention

## Abstract

Although psychological interventions might have a tremendous potential for the prevention of mental health disorders (MHD), their current impact on the reduction of disease burden is questionable. Possible reasons include that it is not practical to deliver those interventions to the community *en masse* due to limited health care resources and the limited availability of evidence-based interventions and clinicians in routine practice, especially in rural areas. Therefore, new approaches are needed to maximize the impact of psychological preventive interventions. Limitations of traditional prevention programs could potentially be overcome by providing Internet- and mobile-based interventions (IMIs). This relatively new medium for promoting mental health and preventing MHD introduces a fresh array of possibilities, including the provision of evidence-based psychological interventions that are free from the restraints of travel and time and allow reaching participants for whom traditional opportunities are not an option. This article provides an introduction to the subject and narratively reviews the available evidence for the effectiveness of IMIs with regard to the prevention of MHD onsets. The number of randomized controlled trials that have been conducted to date is very limited and so far it is not possible to draw definite conclusions about the potential of IMIs for the prevention of MHD for specific disorders. Only for the indicated prevention of depression there is consistent evidence across four different randomized trial trials. The only trial on the prevention of general anxiety did not result in positive findings in terms of eating disorders (EDs), effects were only found in *post hoc* subgroup analyses, indicating that it might be possible to prevent ED onset for subpopulations of people at risk of developing EDs. Future studies need to identify those subpopulations likely to profit from preventive. Disorders not examined so far include substance use disorders, bipolar disorders, stress-related disorders, phobic disorders and panic disorder, obsessive–compulsive disorder, impulse-control disorders, somatic symptom disorder, and insomnia. In summary, there is a need for more rigorously conducted large scale randomized controlled trials using standard clinical diagnostic instruments for the selection of participants without MHD at baseline and the assessment of MHD onset. Subsequently, we discuss future directions for the field in order to fully exploit the potential of IMI for the prevention of MHD.

## Introduction

Mental health disorders (MHD) are highly prevalent, with estimated lifetime and 12 month-prevalence rates, ranging across countries between 18.1–36.1 and 9.8–19.1%, respectively (1). MHD are one of the leading causes of disability (2) and associated with an immense disease burden such as poorer quality of life of sufferers and their loved ones, an increased risk of developing chronic physical conditions and related mortality (3–5). The economic burden of these disorders is enormous, including substantial economic costs, reduced workforce participation, occupational impairment, and lost productivity (6–8).

In the past decades, a variety of interventions have been developed to treat MHD for which efficacy has been demonstrated in a large number of randomized trials (9, 10). However, even assuming the hypothetical scenario of 100% coverage and compliance to evidence-based treatments, approximately only 28% of the disease burden attributable to MHD could be averted (11). In fact, less than half of the individuals with a MHD are recognized and treated (12). Therefore, attention has increasingly been focused on the prevention of MHD.

Preventive interventions can be classified as universal interventions, directed at the whole population; selective interventions, directed at individuals with specific risk factors for the development of a MHD; or as indicated preventive interventions, directed at individuals in the prodromal stage of a disorder, who do not yet fulfill the criteria for a full blown disorder but experience subclinical symptoms (13).

Emerging evidence indicates the potential of psychological interventions for the prevention of MHD. For example, in a recent meta-analysis, van Zoonen and colleagues found psychological interventions aiming to prevent major depressive disorders (MDDs) to reduce the incidence by approximately 22% (14). Results from another review found that cognitive behavioral indicated preventive interventions reduced the transition to psychosis with a risk ratio of 0.54 (95%-CI: 0.34–0.86) (15); encouraging evidence from a limited number of randomized controlled trials is also available, for example, for the prevention of eating disorders (EDs) (16) and tobacco use (17), whereas the efficacy for other disorders such as interventions to prevent anxiety (18) is not yet established.

Although psychological interventions might have a tremendous potential for the prevention of MHD, their current impact on the reduction of disease burden is questionable. Possible reasons include that it is not practical to deliver those interventions to the community *en masse* due to limited health care resources and the limited availability of evidence-based interventions and clinicians in routine practice, especially in rural areas. Therefore, new approaches are needed to maximize the impact of psychological preventive interventions.

Limitations of traditional prevention programs could potentially be overcome by providing Internet- and mobile-based interventions (IMIs). This relatively new medium for promoting mental health and preventing MHD introduces a fresh array of possibilities, including the provision of evidence-based psychological interventions that are free from the restraints of travel and time and allow reaching participants for whom traditional opportunities are not an option.

Internet- and mobile-based interventions have been shown to be effective in clinical populations, including the treatment of depression (19–21), anxiety (20, 22, 23), alcohol use (24), and sleep disorders (25). However, evidence for their effectiveness in preventing the incidence of MHD is much less documented.

This article provides an introduction to the subject and narratively reviews the available evidence for the effectiveness of IMIs with regard to the prevention of MHD onsets. Subsequently, we will offer some suggestions regarding the direction of future research in this field.

## Characterizing IMIs

**Figure 1:**
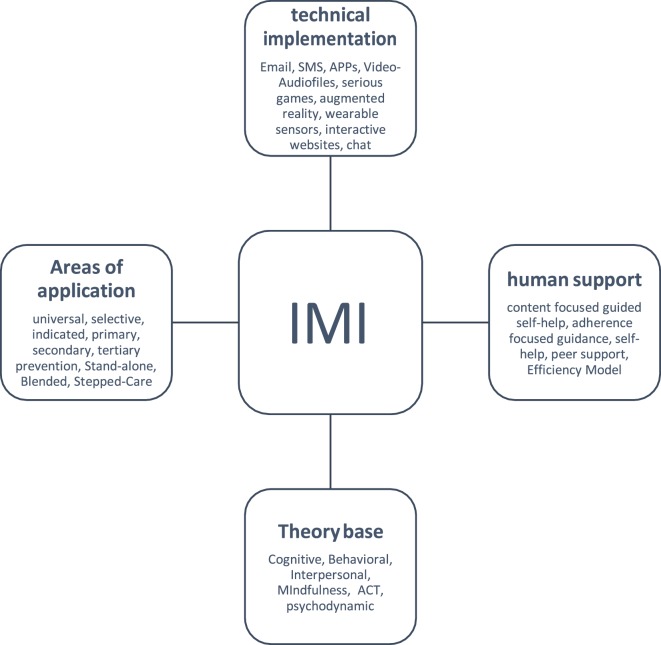
Characteristics of Internet- and mobile-based interventions (IMIs).

The possibilities to use IMIs for the prevention of MHD range from mobile-based apps for the monitoring of health behavior and stand-alone self-help interventions to supplemental elements integrated in conventional on-site psychological interventions (blended concepts). One common element of such interventions is that emotional, cognitive, and behavioral processes are modified and their generalizations to users’ daily lives promoted using established psychological techniques (26). IMIs can be categorized in regard to their use of technology, the extent of human support, the theoretical basis, and with respect to their areas of applications and indications (Figure 1).

### Technical Implementation

For the implementation of IMIs, numerous technical possibilities are applicable. These range from (1) the provision of evidence-based strategies as interactive self-help lessons; (2) e-mail, chat, or video-based sessions (27); (3) virtual reality for exposure interventions (28); (4) serious-games, in which psychological strategies are trained in the context of a computer game (29); (5) the use of automated memory, feedback, and reinforcement interventions, for example, through apps, e-mails, text messages, or short prompts, which support the participant in incorporating intervention content into everyday life; to (6) sensors and apps for monitoring health behavior such as physical activity, which can be used to support the learning process (30).

### Theoretical Basis

Due to their distinctive structured nature, standardization, and focus on the training of strategies and specific behavior, IMIs are particularly suited for techniques that target changes in thoughts and behaviors (26). These include well-researched cognitive, behavioral, and interpersonal interventions. Approaches such as mindfulness-based methods, acceptance and commitment therapy, or psychodynamic approaches, which are already used within some clinical IMIs (31–35), also have the potential for application to preventive IMIs.

### Human Support

As a basic principle, IMIs can be implemented with varying degrees of human support. The current most commonly used method is the so-called “guided self-help,” in which evidence-based content is usually provided as self-help material so that the participants can perform most tasks independently. An accompanying coach then regularly gives feedback on the completed exercises. Fostering adherence to the content of the intervention is usually the main aim of human support, rather than the delivery of new therapeutic techniques that go beyond the content of the current lesson (36, 37). The main task of the coach is to clarify any comprehension questions, provide feedback on solved problems and progress, and to encourage participants to continue to work on themselves. For this to happen, communication can happen either synchronously (per chat or video) or asynchronously (for example, per e-mail), the latter of which is more commonly used, and normally takes a few minutes to a few hours (1–3 h) per participant and intervention. For the participant, the processing of self-help material, execution and repetition of exercises, as well as correspondence with a coach can, however, be very intense and require a much greater time investment than that of the supporting coach (38). The combination of self-help material with minimal human support *via* the Internet thereby increases empowerment of the participants and the degree of self-directed coping while maximizing the efficiency of the accompanying coach. Irrespective of location, asynchronous contact and time-independent communication results in increased flexibility and autonomy for both participants and prevention workers.

An “Efficiency Model of Support” (39) has been recently put forward to contribute to the development of a taxonomy of elements involved in guided interventions, such as type, quantity, timing, quality, and cost of human support. A commentary on that article (40) suggests that it may be useful to categorize contemporary interventions into four major types: traditional face-to-face (FTF) interventions, FTF interventions augmented by behavior intervention technologies (BITs), BITs augmented by human support, and BITs that are fully automated.

### Areas of Application

Applications of IMIs range from mental health promotion and mental disorder prevention to full treatment of mental disorders and interventions to reduce relapse or recurrence. In the fields of psychological health promotion and prevention, IMIs are considered a promising approach for increasing the accessibility of evidence-based psychological techniques to people on a larger scale due to their low threshold for accessibility, location and time independence, and anonymous usability (41). IMIs can be used in the prevention of mental disorders either as a stand-alone approach, as part of a stepped-care approach or as an integrated element of a preventive intervention consisting of online and conventional on-site sessions (blended).

As a *stand-alone measure*, IMIs increase the reach of effective psychological interventions. Telehealth interventions (live therapy online) can transcend space. IMIs can transcend space and time. For example, the temporal and spatial independence of IMIs facilitates access to evidence-based interventions for individuals with limited mobility or those who live in areas with low access to preventive interventions. Populations who are not able to attend appointments during usual visiting hours and, therefore, are not able to attend other on-site FTF options, would then also be able to participate in interventions in the evenings or on the weekend at their own pace. Persons who would have not sought to participate in a preventive intervention due to other individual reasons, such as fear of stigma, would also have access to IMIs. Despite increasing social acceptance of psychological interventions, everything that might be associated with mental problems produce for some individuals a sense of shame, which in itself creates a barrier to the actual use of preventive interventions (39, 40). Moreover, a general problem with preventive interventions lies in the fact that, by definition, the impairment people appropriate for preventive interventions are experiencing is low or not noticeable to them, diminishing their willingness to “invest” into mental health interventions. Hence, the lower the threshold and effort associated with participating in a preventive intervention, the likelier it is that the target group makes actual use of it.

In the combination of IMIs and personal FTF interventions, the so-called *Blended-Concept*, IMIs take over areas that need not necessarily be mediated by a prevention worker, allowing more time during the sessions for FTF psychological process work (26). Prevention workers could, for example, delegate time-consuming routine aspects of the intervention, such as the delivery of psychoeducation to digital tools. In principle, IMIs could also be used to improve FTF interventions by providing exercises for the participant to work on in between the intervention sessions, thereby increasing intervention intensity. Another way in which IMIs could be used to improve the outcome of FTF interventions is by supporting the integration of behavior changes or training of techniques into routine life, thus extending the reach of the psychological intervention into the daily lives of participants. This can be achieved through methods, such as smartphone-based behavioral diaries, sending of messages with ultra-short prompts aimed at training-specific strategies in daily life, or smartphone-based coaches that lead patients through potential anxiety-provoking or other difficult situations.

Furthermore, the objective of most psychological interventions is that participants actively try to integrate new behavior into their daily life and maintain these changes in the long term. IMIs emphasize the active role of the person concerned in this process, thus promoting a sense of empowerment through encouraging them to use their own resources to solve problems. IMIs could be used by people much before there is any need to seek FTF mental health services (and hopefully prevent the need to do so), during times when they are receiving treatment, as adjuncts to FTF care, and after treatment ends, to maintain gains, continue to progress in terms of reaching ever sturdier mental health levels, and to reduce relapse and recurrence.

Within *stepped-care approaches*, the degree of support participants receive are designed according to their actual individual need. In step-up interventions (guided) preventive approaches can be offered, for example, to individuals in the prodromal disease stage (indicated prevention) as a first element in the chain of treatment in order to prevent the transition to the full blown disorder. Further intensive therapeutic support, such as outpatient psychotherapy, then occurs should the patient respond insufficiently to the self-help intervention. Similarly, step-down interventions supplement more intensive therapeutic measures with lower intensity support. For example, IMI-relapse prevention concepts could be offered to patients following an acute treatment in order to stabilize acute treatment effects and thereby prevent relapse and recurrence (42–46).

## Effectiveness of IMI in Preventing the Onset of MHD

While there are currently well over 100 randomized controlled studies on Internet- and mobile-based concepts fostering mental health, only few studies to date have investigated their potential in preventing the incidence or onset of a MHD. In the following section, we will review these studies and thereby only focus on studies that assessed the effects of an IMI on MHD onset (assessed according to diagnostic and statistical manual of mental disorders/ICD criteria) in a sample of adults, adolescents, or children, who is free from an acute mental disorder at baseline. There are further studies on mental health IMIs evaluated in samples with subthreshold mental health conditions. However, these studies were often planned for other purposes than examining disorder onset and limited in their methodological quality (47). Table 1 gives an overview of the reviewed studies, and Tables 2 and 3 are about the characteristics of the reviewed studies and interventions.

**Table 1:** Summary of included randomized controlled trials that assessed MHD onset using categorical ICD/DSM diagnostic criteria.

Study	Prevention type	Disorder	Target group	Program type	Program	Conditions	*N*	Follow-up	Instrument	Results
Buntrock et al. (48)	Indicated	Depression	Adults, subthreshold depression (CES-D > 16) No MDD	Stand-alone	GET.ON mood enhancer 6 weeks CBT, guided self-help for subthreshold depressive symptoms	IG: intervention CG: online-psychoeducation	406	12 months	SCID	MDD onset within 12 months IG: 32% CG: 47% RRR = 39% HR = 0.59 (*p* = 0.002) NNT = 5.9

Christensen et al. (49)	Selective	Depression	Adults, primary insomnia (MINI) and depressive symptoms (PHQ > 3 < 20) No MDD	Stand-alone	SHUTi 6 weeks unguided self-help for insomnia symptoms	IG: intervention CG: attention control	1,149	6 months	MINI	MDD onset within 6 months IG: 0.78% CG: 1.13% ns

Christensen et al. (50)	Indicated	GAD	Adults 18–30, GAD symptoms (GAD-7 > 5) No PD, SP, BDP, schizophrenia, psychosis Not undergoing psychiatric treatment	Stand-alone	iChill 10 weeks unguided iCBT for anxiety symptoms	IG1: unguided iChill IG2: iCHill + phone reminders IG3: iChill + e-mail reminders CG1: attention control website CG2: attention control website + phone reminders	558	6 months	MINI	GAD onset within 6 months Across all IG: 6.7% Across all CG: 4.5% ns

Imamura et al. (51)	Indicated	Depression	Adult workers with self-identified subthreshold depressive symptoms (WHO-CIDI 3.0, self-administered) No MDD	Stand-alone	6 weeks unguided iCBT, manga comic-based intervention for depression, feedback on demand	IG: unguided iCBT CG: e-mail with non-CBT stress-management tips	822	12 months	WHO-CIDI 3.0 self-administered	MDD onset within 6 months IG: 0.8% CG: 3.9% HR = 0.22 RRR = 0.20 (*p* = 0.009) NNT = 32

Lindenberg and Kordy (52)	Universal	EDs	Secondary education students (13–16) No ED diagnosis Not undergoing treatment (ED)	Stepped care	Young E[s]sprit stepped guided intervention (ranging from unguided feedback and self-help, though peer support to individual counseling)	IG: intervention CG: online-psychoeducation	1,667^a^	12 months	LIFE	Any ED onset within 12 months, IG1: 5.9% CG1: 9.6% (*p* = 0.038) HR = 1.67 IG2: 5.6% CG2: 4.8% ns

Taylor et al. (53)	Selective	EDs	College-age women, weight shape concern No diagnosed ED	Stand-alone	Student bodies 8 weeks guided CBT-based self-help treatment	IG: intervention CG: wait list control	480	24 months	EDE	Any ED onset within 24 months IG: 10% CG: 5% ns

Thompson et al. (54)	Indicated	Depression	Adult epilepsy patients, subthreshold depression (CES-D > 8, <27, PHQ-9)	Stand-alone	UPLIFT 8 weeks Internet-/telephone-delivered mindfulness cognitive therapy-based intervention	IG: UPLIFT CG: wait list control	128	8 weeks	PHQ-9	MDD onset within 8 weeks IG: 0% CG: 10.7% (*p* = 0.028)

Holländare et al. (55, 56)	Indicated, relapse prevention	Depression	Adults, MDE in the past 5 years, subthreshold depression (MADRS-S > 7, <19) Not undergoing treatment No BDP, psychosis, addiction	Stand-alone	10 weeks guided Internet-based CBT self-help intervention for depressive symptoms	IG: intervention CG: TAU CG	84	6 and 24 months	SCID	MDD onset within 6 months IG: 10.5% CG: 37.8% within 24 months IG: 13.7% CG: 60.9% (*p* = 0.001) HR = 0.16

Bauer et al. (57)	Selective, relapse prevention	Transdiagnostic	Adult discharged stationary patients No psychotic symptoms	Stepped-care	12–15 weeks Internet-based guided non-manualized chat intervention	IG: chat intervention CG: TAU CG	152	12 months	LIFE	Any DSM disorder onset within 52 weeks IG: 22.2% CG: 46.5% (*p* < 0.1)

Taylor et al. (58)	Selective	EDs	Young adult women, weight/shape concerns (WCS ≥ 47), eating-related teasing, depression or non-clinical compensatory behavior No diagnosed ED	Stand-alone	Image and mood 10 weeks guided CBT-based self-help treatment	IG: intervention CG: wait list control	185	24 months	EDE	ED onset within 24 months IG: 24% CG: 31% ns HR = 0.73

*Universal—universal prevention. Interventions directed at the whole population; selective—selective prevention. Interventions directed at individuals with specific risk factors for the development of a MHD; indicated—indicated prevention. Interventions directed at individuals in the prodromal stage of a disorder; relapse prevention—interventions aiming to reduce relapse and recurrences after first onset*.

*^a^Two waves (wave 1: *N* = 896, wave 2: *N* = 771)*.

*CES-D, Center for Epidemiological Studies Depression Scale; CBT, cognitive behavioral therapy; IG, intervention group; CG, control group; SCID, structured clinical interview for DSM disorders; MDD, major depressive disorder; RRR, relative risk reduction; HR, hazard ratio; p, level of significance; NNT, number needed to treat; MHD, mental health disorders; MINI, Mini-International Neuropsychiatric Interview; PHQ, patient health questionnaire; GAD, generalized anxiety disorder; PD, panic disease; SP, social phobia; BDP, borderline personality disorder; WHO-CIDI, World Mental Health Composite international Diagnostic Interview; EDs, eating disorders; LIFE, longitudinal interval follow-up evaluation; TAU, treatment as usual; DSM, diagnostic and statistical manual of mental disorders*.

**Table 2:** Target conditions addressed by studies investigating the effectiveness of Internet- and mobile-based interventions on mental health disorders onset.

Study	Unipolar depression	Bipolar	Eating disorders	Psychosis	Addiction	Stress-related disorders	Phobic disorders	Panic disorders	Obsessive–compulsive disorders	Generalized anxiety	Impulse control disorders	Insomnia	Transdiagnostic
Buntrock et al. (48)	X												
Christensen et al. (49)	X												
Christensen et al. (50)										X			
Imamura et al. (51)	X												
Lindenberg and Kordy (52)			X										
Taylor et al. (53)			X										
Thompson et al. (54)	X												
Holländare et al. (55, 56)	X												
Bauer et al. (57)													X
Taylor et al. (58)			X										

Total number of studies	5	0	3	0	0	0	0	0	0	1	0	0	1

**Table 3:** Characteristics of studies and interventions that investigated the effectiveness of Internet- and mobile-based interventions on mental health disorders onset.

Study	Target group			Prevention type			Media type		Program features			Cost-effectiveness evaluated	Reported potential negative effects	Type of human support	
							
	Children	Adolescent	Adults	Universal	Selective	Indicated	Internet	Mobile	Sensors	Wearables	Algorithms			Guided	Unguided
Buntrock et al. (48, 59)			X			X	X	X				X		X	
Christensen et al. (49)			X		X		X								X
Christensen et al. (50)			X			X	X								X
Imamura et al. (51)			X			X	X								X
Lindenberg and Kordy (52)		X		X			X							X	
Taylor et al. (53)			X		X		X							X	
Thompson et al. (54)			X			X	X							X	
Holländare et al. (55, 56)			X			X	X							X	
Bauer et al. (57)			X		X		X							X	
Taylor et al. (58)			X		X		X							X	

Total number of studies	0	1	9	1	4	5	10	1	0	0	0	1	0	7	3

### Indicated Prevention

#### Depression

Three studies have evaluated the effects of Internet-based approaches with regard to the primary prevention of depression. Buntrock et al. recently published a trial on the effects of an Internet-based indicated prevention stand-alone intervention (41, 48, 60). They randomized 406 adults with subclinical symptoms of depression who did not fulfill the criteria for MDD in the last 6 months to either a 6-week guided Internet-based cognitive behavioral intervention or to an online passive psychoeducation intervention. The intervention group (IG) included behavioral activation and problem solving as core intervention components; in addition, participants were able to choose among several different optional modules (e.g., sleep hygiene/sleep restriction, progressive muscle relaxation, and rumination techniques). In addition to the Internet-based self-help module, a text message coach sent a set of “tiny tasks” to the participant’s mobile phone in order to foster the application of intervention techniques in daily life. In the IG, 32% of the participants experienced a MDD during the 12 months of follow up, whereas 47% in the control group (CG) did. Cox regression analyses controlling for baseline depressive symptom severity showed a hazard ratio (HR) of 0.59 indicating a 41% reduction in the risk for developing a MDD with a number need to treat to avoid one new case of MDD of 5.9. It is important to note that, in this trial, it was not assessed whether participants had a prior history of MDD. Hence, future studies are needed to investigate whether the effects count both for first incidence and subsequent onset of MDD.

In a randomized crossover trial, Thompson and colleagues evaluated an 8-week Internet or telephone-delivered mindfulness-based stand-alone intervention in 64 adult epilepsy patients with subthreshold depressive symptoms. They found that the incidence of MDD episodes, assessed *via* self-report from baseline to interim assessment was significantly lower in the intervention condition (0.0%) than in treatment as usual (TAU) (10.7%) condition 8 weeks following randomization. Half of the participants were assigned to receive the intervention *via* web, half *via* telephone, but the authors did not find any differences between the two different forms. Although future studies with longer follow-up periods, larger sample sizes and observer-based clinical interviews are clearly needed to determine the potential of the approach for the prevention of depression, this trial is an example of the potential of Internet-based approaches.

Imamura and colleagues (51) evaluated an Internet-based indicated prevention program with workers who self-identified as having depressive symptoms but not fulfilling the diagnostic criteria for MDD. They randomized 822 workers either to a 6-week, Internet-based cognitive behavioral program delivered in a comic-form or to a wait list CG. The CBT components of the program included self-monitoring, cognitive restructuring, assertiveness, problem-solving, and relaxation. Results showed significantly lower incidence of MDE at the 12-month follow-up, with 0.8 and 3.9% of the experimental and control participants, respectively, experiencing a MDE. This corresponds to a HR of 0.22 and a numbers needed to be treated of 32 in order to prevent one case of major depression. However, the results need to be interpreted with caution as the diagnosis of MDD had been established only on the basis of a self-report instrument [World Mental Health Composite International Diagnostic Interview (WHO-CIDI) self-administered] and not using standard clinician/expert-based diagnostic instruments.

#### Anxiety Disorders

The only trial we are aware of that evaluated the effects of an IMI on anxiety disorder onset is an indicated prevention of general anxiety disorder trial conducted by Christensen et al. (50). They evaluated three different versions of iChill, a 10-week Internet-based cognitive behavioral intervention for anxiety symptoms (without any reminders, phone reminders, and e-mail reminders) compared to two attention placebo CG (interactive, attention-matched, Internet-based placebo control program “Healthwatch” with/without phone reminders) in 558 young adults (age 18–30) with general anxiety symptoms who did not meet the criteria for an anxiety disorder.

Generalized anxiety disorder onset at 6-month follow up was 6.7% across all IGs, and 4.5% across both CGs, a difference that was not statistically significant.

### Selective Prevention

#### Depression

Christensen and colleagues evaluated the effects of a 6-week unguided fully automated Internet-based intervention for sleeping problems (SHUTi) with regard to the prevention of major depressive episodes (49, 61). They randomized 1,149 adults with primary insomnia and depressive complaints who did not fulfill the criteria for a major depression to either SHUTi or to Healthwatch. Although large effects in the SHUTi group on insomnia complaints and a lower depression symptoms on the patient health questionnaire-9 at 6 weeks and 6 months compared with Healthwatch were found, the intervention was not superior with regard to the effect on diagnosis of MDD assessed with the Mini-International Neuropsychiatric Interview. However, only ~4% percent of the total sample developed a major depression during the 6-month follow up, making it difficult to detect any preventive effects, even at such a large sample size. Hence, future studies on the longer term follow-up data of this trial (12/18 months) are needed in order to conclude whether this approach is indeed promising with regard to the prevention of major depressive episodes.

#### Eating Disorders

At least two randomized trials have been conducted to date that focused on the selective prevention of ED onset. Taylor and colleagues evaluated the effects of an online cognitive behavioral selective preventive intervention (StudentBodies) on ED incidence compared with a wait list control condition over 3 years in a sample of women with a body mass index >24 and any baseline compensatory behaviors (53). Although they found the intervention to be efficacious in reducing high weight/shape concerns over a period of 24 months and lower ED incidence rates in the intervention (5%) group compared to controls (10%), the difference in ED onset was only significant in a subgroup of individuals, recruited and treated at one particular trial site. However, they identified in subsequent analyses specific risk factors associated with ED onset [i.e., comments/teasing about eating from a teacher, coach, or sibling and lifetime depression (62)]. Based on these findings, they adapted the IMI to target these specific risk factors and subsequently evaluated this intervention (image and mood) in 185 young adult women (age 18–25) with elevated weight/body shape concerns, eating-related teasing, depression and compensatory behavior compared to a wait list-CG. Although ED onset rates within a 24-month follow up was 27% lower in the intervention compared to the CG, this did not reach the level of statistical significance. Significant lower onset rates were, however, found in a subgroup with the highest body shape concerns, onset (20 vs. 42%, number needed to treat = 5), indicating that it is possible to prevent EDs in very high-risk samples. Another study on StudentBodies showed promising effects on subthreshold ED onset, but did not investigate the effects on full blown disorder onset (63).

### Universal Prevention

To the best of our knowledge, only one study has been published to date evaluating a universal prevention approach with regard to MHD onset. Lindenberg and Kordy (52) evaluated a universal approach for the prevention of EDs in secondary education students (age 13–16) with no prior ED diagnosis and not undergoing treatment for any ED. Young E[s]sprit is a stepped program tailored to the individual risk of the participant, with elements ranging from screening and tailored risk feedback plus recommendations for specific self-help modules, through monitoring of risk behavior and symptoms and synchronous group and individual online chats up to individual FTF counseling. Schools including a total of 1,667 adolescents were cluster-randomized in two waves to receive either Young E[s]sprit or an online-psychoeducation intervention. Results showed significantly reduced ED onset rates in the IG compared to control schools in the first wave (intervention: 5.6%, controls: 9.6%) but the second wave (intervention: 5.6%, controls: 4.8%) did not yield significant differences in the overall analyses.

### Relapse Prevention

By now, quite a few studies have investigated the effects of IMI as a relapse prevention intervention (42–45, 64, 65), and at least two of these also investigated the effects of an IMI on the prevention of MHD relapse.

Holländare and colleagues (55, 56) evaluated a 10-week guided self-help cognitive behavioral intervention for the prevention of relapse in 84 partially remitted depressed adults with at least one previous depressive episode in the past 5 years compared to a no-treatment CG. Six (intervention 5%, controls: 37.8%) and 24 months (intervention: 13.7%, controls: 60.9%) following randomization they found lower rates of relapse in the IG compared to the CG with a HR for time to relapse within 24 months of HR 0.16 in favor of the IG.

Bauer and colleagues (57) investigated the effects of a synchronous transdiagnostic non-manualized Internet-chat group as a stepped-care intervention following inpatient psychotherapy of MHD compared to TAU in 152 adults. They found the chat group to significantly reduce the risk for relapse with 46.5 and 22.2% of the participants experiencing a relapse within 1 year of inpatient discharge in the control and IG, respectively.

## Future Directions for the Field

Although significant strides have been made in recent years regarding the development of effective IMIs for the prevention of MHD, there are several important directions for future research.

## The Need for More Rigorously Conducted Large-Scale Randomized Controlled Trials

First, the number of randomized controlled trials that have been conducted to date is very limited and so far it is not possible to draw definite conclusions about the potential of IMIs for the prevention of MHD for specific disorders. Only for the indicated prevention of depression there is consistent evidence across four different trials (three primary prevention trials aiming to reduce first incidence, one relapse prevention trial). However, only one primary prevention trial (48) and the relapse prevention trial (56) used standard diagnostic procedures, while the other two trials relied only on self-report questionnaires for onset identification. The only trial on the prevention of general anxiety did not result in positive findings. In terms of EDs, effects were only found in *post hoc* subgroup analyses, indicating that it might be possible to prevent ED onset for subpopulations of people at risk of developing EDs. Future studies need to identify those subpopulations likely to profit from preventive IMIs. It should be noted that two of the five successful prevention trials with positive findings (54, 56) were based on very small sample sizes, whereas several much larger trials did not find positive results. Disorders not examined so far include substance use disorders, bipolar disorders, stress-related disorders, phobic disorders and panic disorder, obsessive–compulsive disorder, impulse-control disorders, somatic symptom disorder and insomnia. However, it is of note that there is quite substantial evidence for the effectiveness of health behavior change IMIs regarding the reduction of problematic alcohol consumption (24), improving sleep (66–68), reducing work-related stress (36, 69–71), all of which might be useful as MHD prevention IMIs as well. In summary, there is a need for more rigorously conducted large-scale randomized controlled trials using standard clinical diagnostic instruments for the selection of participants without MHD at baseline and the assessment of MHD onset.

## Assessing Diagnostic Status at Baseline and Follow-Up

Second, one general problem of prevention trials is that one needs very large sample sizes in order to be able to detect existing differences between groups, as transition rates to full blown disorders tend to be low during follow-ups of typical length of controlled studies even in high-risk groups. This problem is (could be) true of many of the studies reviewed above (49–51, 53, 54, 58). Given the low chance of positive findings in a trial that is not primarily designed nor powered to detect such findings, it is understandable that many prevention researchers abstain from assessing diagnostic status. However, even without positive findings such information would be very valuable for the field and we would like to encourage researchers to assess these data. Diagnostic status data could help to obtain the necessary information on the transition rates of the target population and generate hypotheses on the size of the effect on MHD onset, which are both necessary to design and power subsequent trials adequately. Moreover, if several of such smaller trials would be conducted, the data of these trials could be combined using individual participant data meta-analytic techniques (72, 73). By collecting and pooling the primary data of individual trials, multiple underpowered trials can contribute to a large enough pooled sample size with sufficient power to examine effects on the incidence of MHD as well as analyzing patient subgroups and other effect modifying variables (74). If conducting observer-based clinical interviews is too expensive, web-based self-administered version of instruments such as the WHO-CIDI could be a low-cost alternative (75).

## Examining Different Prevention Settings and Approaches

Provided that preventive IMIs are transferable to clinical praxis, we further need to establish the best way to implement preventive IMIs into our health care systems. Integrating IMIs as a first step of stepped-care approaches might be one promising way. Prevention IMIs for MHDs might also easily be integrated in already existing prevention programs (blended MHD prevention). That this would be worthwhile is supported by recent meta-analytic findings, indicating that traditional FTF interventions profit from providing additional IMI-components (76). Lindhiem and colleagues showed in their systematic review on 10 RCTs that a mobile component as a supplemental element in psychological interventions (e.g., SMS to support behavior changes between therapy sessions) considerably increase the effectiveness of these interventions compared to the respective strictly on-site interventions (SMD = 0.27) (76). IMIs also offer the unique potential of reaching people who would not access preventive mental health care *via* the established channels delivery, either because they do not utilize the available offers or because they do not feel comfortable discussing their mental health issues with their general practitioners and mental health specialists. Thus, we should also think about implementing preventive MHD–IMIs in a way that allows people to self-refer to IMIs [10]. This might, for example, be achievable by providing preventive IMIs *via* websites of established associations or as a direct prevention offer from health insurance companies, which would probably make it necessary for several countries to think about alternative financing health care models to integrate preventive IMIs in the best possible way. However, an approach just aimed at the GP and health insurance companies might be too narrow to exploit the potential of preventive services, as the majority of the target population might not use them. Preventive IMIs should be, therefore, I think that preventive interventions should be delivered through multiple channels that have “natural” possibilities to engage people, such as schools (they can reach all students), universities and colleges, pregnant women (because they all receive prenatal care), patients with general medical disorders, but also, for example, companies.

## Studies in Children, Adolescents, and Young Adults

Most of the trials that have been conducted up to this point have included only adults as participants. Although the general potential of IMIs to foster mental health in children and adolescents has been documented (20), only one study has investigated the effects of a prevention IMI, delivered as a universal preventive approach, on MHD onset (52). Given that ~75% of all MHD have their onset before the age of 25 (77), future studies should explore the potential of IMIs for preventing the first incidence in children, adolescents and young adults. That this is possible using a psychological intervention has been shown, for example, for depression (78). An interesting development in this field is a shift away from traditional computerized and browser-based interventions to mobile-based smart phones interventions. Using the most up-to-date technology and/or access paths for providing mental health prevention might increase the interventions attractiveness and user-friendliness in this modern technology oriented age group. However, evidence from mobile-based interventions and other mode of deliveries is yet scarce and future studies are needed to explore their potential. One of these other possibilities are serious games or even augmented reality interventions, that go beyond what can be done in typically “talking” interventions likely to attract children and adolescents. The potential of serious games to foster mental health has been shown, for example, in the field of depression (29, 79), but yet there is no evidence regarding the prevention of MHD onset, with one trial currently being conducted (80). The use of augmented reality has to the best of our knowledge not yet been explored. One general problem with psychological interventions for the prevention of MHD in children and adolescents is that one needs parental consent, which is difficult to obtain online in a reliable manner, and which can be seen as barrier to reach these high-risk groups.

Although many MHD already have their initial incidence before college matriculation (81), college entry might nevertheless be a very promising point in time to deliver preventive psychological interventions. College entry allows screening of the whole college student population, identification of those at risk for development of mental health problems, and subsequent offers of targeted preventive interventions. These might be disorder-specific prevention IMIs or trainings focusing on missing skills and competencies (e.g., procrastination, limited social competencies, and low self-efficacy) as well as other known risk factors for developing mental disorders. Similarly, entering vocational schools and the working environment might be a possibility to establish mental health screening and subsequent mental health trainings. In this context, where freshmen might be anxious about disclosing mental health issues, the possibility of IMIs being provided anonymously can be regarded as one substantial advantage over other occupational and college mental health management programs.

## Evaluating the Role of Human Support in Preventive Interventions

After development of an IMI, ongoing costs are directly related to guidance time. Hence, evaluating and comparing the effectiveness of interventions with different guidance formats is of particular importance. In the present review, only three of ten trials examined an unguided intervention, of which only one found significant, but very small effects on disorder onset. This is in line with previous findings from the clinical field that indicate that IMIs with human support have a significantly greater success than IMIs without therapeutic support (82–84). However, unguided IMIs might still produce larger effects at population level with regard to the reduction of disease burden, as more individuals could be reached at a given budget (85). A recent review on the cost-effectiveness of IMIs for improving mental health problems suggested that guided IMIs might be more cost-effective than unguided IMIs despite their higher intervention costs per participants (86). Hence, there is a need for studies that compare not only the cost-effectiveness of guidance vs. no guidance in randomized trials but also different intensities and forms of guidance [e.g., guidance concept with individual feedback on the completed exercises, i.e., content feedback, vs. feedback aiming only to increase the adherence to the intervention (37)]. Moreover, the type of human support might not only have an impact on the effectiveness of interventions but also on the willingness to use such interventions. Given that the effects of interventions on a population level also depend on the acceptance and the reach of the target population, studies should address both effectiveness and reach of IMIs with different forms of guidance.

## Cost-Effectiveness Studies

Implementing preventive IMIs into our health care system might be a promising strategy regarding the cost-effectiveness of the health care system as has been recently estimated on the basis of a Markov-model study (87). However, evidence from randomized controlled trials is still scarce when it comes to IMIs for the prevention of MHD Studies indicated that guided Internet interventions for depression, anxiety, sleeping problems, smoking cessation, and alcohol consumption have favorable probabilities of being more cost-effective when compared to controls (86, 88). However, these studies were mainly directed at the treatment of mental health problems, and only one study has to the best of our knowledge been published so far, that investigated the cost-effectiveness of an IMI with regard to the prevention of MHD onset (59). Two ongoing studies might provide first results in the near future (41, 46).

## Possible Adverse Effects of MHD Prevention IMIs

As with any other method, it is important to take into account the limitations and risks are involved with IMIs alongside all of the potential benefits of the procedure. At this stage, however, reliable empirical information showing negative effects of preventive IMIs has been very limited (89) and also in the present review, none of the identified studies reported results on potential negative effects.

Potential risks and negative effects include, depending on the concept, the following points, among others: (1) limited ability to timely identify patients prone to self-injury, for example, in relapse prevention interventions; (2) the development of reduced health-related self-efficacy if participants are not successful with a stand-alone IMI; and (3) the development of negative attitudes of non-responders toward psychological interventions in general and as a result a reduced willingness to utilize mental health care in case of MHD onset. Possible negative effects of such interventions cannot be ruled out at present, which counts to a similar degree also to classical FTF psychological interventions. There is an urgent need for further research.

One “cautionary tale” that has been published involves a secondary analysis of the impact of a mood management intervention embedded in an online smoking cessation randomized control trial. Participants came to the online site in order to stop smoking. Half of the sample was randomly assigned to a cognitive behavioral mood management intervention. The authors wondered if smokers at risk for major depressive episodes (defined as having subthreshold levels of major depression symptoms at baseline) who had been randomly assigned to receive the mood management intervention had lower incidence of major depressive episodes at follow up, thus showing a preventive effect. The results showed that the incidence was actually significantly greater for the group assigned to the mood management condition (90). The authors speculate that being assigned to a mood management intervention when one is not looking for such might make a participant more aware of depressive symptoms, thus increasing their self-report scores at follow-up assessments. They then examined a subsequent study in which participants in a similar online smoking cessation trial could freely choose (rather than being randomly assigned to) the elements of the interventions provided, including the same mood management intervention. In this study, participants screening positive for major depression were more likely to choose the mood management intervention, and, if they did so, were more likely to quit smoking (91).

These studies suggest that providing participants with a choice of interventions may be preferable to assigning them interventions that they did not choose and that participants are likely to choose appropriate interventions for themselves.

## Make Use of the Technological Potential of IMIs

All of investigated interventions were delivered over the Internet, only one study additionally used the mobile phone to facilitate the transfer of learned skills in daily life routine (48). None of the studies were delivered mobile only and none of the interventions’ used smartphone sensors, wearables, or artificial intelligence algorithms. It seems that the field so far focused mainly on delivering psychological intervention as a (guided) self-help format through the Internet, without making full use of the technological potential of such approaches. Artificial intelligence algorithms based on user behavior might bare a great potential for supporting participants to change behavior, for example, in form of just-in-time adaptive intervention (92). However, their potential with regard to preventing MHD onset still needs to be proven and should be investigated in future studies.

## Development of Multivariate Prediction Algorithms to Identify People at Risk and Match Participants to Interventions

One general problem in the field of prevention is identifying the right people to target with specific preventive interventions. Many, if not most, individuals displaying single risk factors stay disease free without intervening, which has also occurred in most of the trials reviewed above. The combination of several specific risk factors might be a promising strategy toward overcoming this problem (93), and has been utilized in some first trials (58). Recent advantages in the field of precision medicine and machine-learning techniques might further help to identify the people at highest risk who might profit from a preventive intervention. There have been first studies in the mental health field predicting MDD onset (94, 95) in the general population, general anxiety and panic onset in general practice attendees (96) panic recurrence (97), or recurrence of suicidal ideation (97). A next necessary step in the field is to develop and validate such algorithms further for different populations and then, subsequently, test whether applying these risk prediction algorithms with subsequent preventive interventions is effective in reducing incidence of MHD in these high-risk groups.

The use of supervised machine-learning methods could also be used to explain the heterogeneity in intervention response (98, 99) in order to finally match individual to specific interventions. The necessary large sample sizes to develop such prediction equations are feasible to obtain with scalable IMIs.

A high-risk group that has been noticeably absent from preventive IMI trials is the group of individuals with genetic markers associated with mental disorders. A recent study (100) suggests a method that could combine genetic research with IMI depression prevention research. A massive sample collected by the genetic screening company was used to identify 15 genetic loci associated with risk of major depression: 75,607 individuals reporting clinical diagnosis of depression were compared to 231,747 individuals reporting no history of depression. A replication data set (45,773 cases and 106,354 controls) was then used to confirm the findings.

This very large sample could be the basis for an interesting set of depression prevention studies. Subgroups of individuals who have no history of depression but have the genetic markers associated with risk could be recruited for randomized trials testing promising IMIs. Such studies could contribute to prevention science in several ways by helping to confirm whether individuals with the markers actually have higher risk for developing major depressive episodes, particularly when confronted with stressful life events. They could also determine prospectively which of the 15 loci yield the greatest risk and help determine whether interventions that can be conducted online can reduce incidence in individuals with genetic loading for depression.

## Targeting Underlying Risk and Protective Factors in Individually Tailored Interventions

The use of IMIs allows the provision of tailored interventions on large scale to a degree unlikely ever to be implementable using traditional FTF approaches. So far, most studies evaluated standardized interventions based on CBT, including standard packages (e.g., cognitive restructuring, behavioral activation, relaxation in a depression prevention intervention, etc.) for all participants regardless of the individual specific risk or protective factors, as well as intervention preferences. One potential next step for the field would be to develop and provide intervention modules that target specific underlying risk or protective factors (e.g., rumination, emotion regulation, social skills, experiential avoidance, compensatory behavior anxiety sensitivity, and physical activity) and tailor the combination of these modules based on the individual risk and need profiles of the participant. However, whether such an approach is superior to far more simple to develop and maintain standardized interventions that provide non-tailored to all participants is an empirical question that has yet to be adequately addressed.

## Strategies to Increase Reach and Utilization of Available Interventions

The impact of evidence-based preventive intervention on population level incidence heavily depends on the acceptance and use of such interventions in the target population (101). As the field of MHD prevention continues to make progress in identifying programs that yield positive effects, it will be important to develop and evaluate effective and cost-effective strategies to disseminate evidence-based programs. Hence, there is a need for studies that investigate potential obstacles in order to subsequently develop and evaluate strategies for overcoming them. For example, Ebert, Baumeister, and colleagues evaluated acceptance—facilitating interventions that address potential barriers for acceptance of IMIs in different target populations in a series of randomized controlled trials (102–104). Moessner and colleagues evaluated the effectiveness and cost-effectiveness of different school-based dissemination strategies for the prevention and early intervention in EDs (105). However, such experimentally examined strategies to increase the reach of preventive interventions are scarce and need to be conducted more often in order to fully exploit the potential of preventive interventions on the population level.

## Conclusion

Internet- and mobile-based interventions are flexible, technically diverse methods which lend themselves to a variety of application areas. Such approaches have an ability to reach target groups in a way not yet achieved by classical FTF activities. A number of studies have shown that such interventions can be effective in preventing mental disorders. But clearly much more research is needed in order to fully determine the potential of IMIs for substantially reducing the immense disease burden of MHD at the population level.

## Author Contributions

DE drafted the first draft of the manuscript, all authors contributed to the further writing of the manuscript.

## Conflict of Interest Statement

The authors declare that the research was conducted in the absence of any commercial or financial relationships that could be construed as a potential conflict of interest. The reviewer KW and handling editor declared their shared affiliation, and the handling editor states that the process met the standards of a fair and objective review.
